# Efficacy of COVID-19 Vaccines in Patients with Hematological Malignancy Compared to Healthy Controls: A Systematic Review and Meta-analysis

**DOI:** 10.31662/jmaj.2023-0171

**Published:** 2024-04-01

**Authors:** Anindita Das Barshan, Emilie Louise Akiko Matsumoto-Takahashi

**Affiliations:** 1 Graduate School of Public Health, St. Luke’s International University, Tokyo, Japan; 2 Public Health, Dhaka Medical College, Dhaka, Bangladesh

**Keywords:** COVID-19 vaccines, Hematological malignancy, Seroconversion, Immunogenicity, Systematic review

## Abstract

**Background:**

The possibility of developing a severe coronavirus infectious (COVID-19) disease caused by severe acute respiratory syndrome coronavirus 2 (SARS-CoV-2) has increased, particularly in patients with hematological malignancies. These patients are more likely to produce less antibody protection due to the immunocompromised nature of the disease and the anticancer treatments. Therefore, the present systematic review intended to evaluate the seroconversion rate of COVID-19 vaccines in patients with hematological malignancies compared with healthy controls.

**Methods:**

A comprehensive systematic search was conducted in Medline via PubMed, EMBASE, and the World Health Organization COVID-19 Research Database, as well as other searches (i.e., reference list from article search and manual searches), from December 2020 to May 2022. The outcome of interest included estimating the seroconversion rates following COVID-19 vaccination in patients with hematological malignancies and comparing them with those in healthy controls. After two-step screening, the data were extracted and the summary measures were calculated using a random-effects model.

**Results:**

A total of 39 articles regarding patients with hematological malignancies were included in the present review. After the first vaccine dose, these patients had considerably lower antibody response rates (37.0%) compared with healthy controls (74.5%). Following the second vaccine dose, the seroconversion rate in patients reached 66.8%, whereas it peaked at 97.9% in the healthy controls following complete immunization. Notably, the BNT162b2 and ChAdOx1 vaccine combination achieved the highest seropositivity rate of approximately 70%. Multiple myeloma, chronic lymphocytic leukemia, and lymphoma were the cancers of interest in most of the studies.

**Conclusions:**

The results of the present study highlighted the comparatively low seropositivity rates in patients with hematological malignancies, with substantial variations in rates across disease groups. The findings emphasize the possibility of additional booster doses for these individuals to enhance their immunity against SARS-CoV-2.

## Introduction

The global spread of COVID-19 caused by SARS-CoV-2 called for an immediate worldwide action to combat its high mortality. The Food and Drug Administration approved an Emergency Use Authorization for the first COVID-19 vaccine on December 11, 2020 ^(1)^. Several vaccines were developed in response, including BNT162b2 (Pfizer-BioNTech), mRNA-1273 (Moderna), AZD1222 (Oxford/AstraZeneca), Ad26.COV2.S (Johnson & Johnson), Sputnik V (Gamaleya), and BBIBP-CorV (Sinopharm), with efficacy values between 60% and 94%, and were generally well tolerated ^(2)^. In the subsequent year, several clinical trials and observational studies assessed the efficacy of the COVID-19 vaccination in healthy individuals. However, few trials included individuals with chronic disease who had relatively safe profiles. Immunocompromised individuals were often excluded from such studies because of vulnerabilities, resulting in a gap in the empirical data for this cohort.

Pertinently, patients with cancer are susceptible to severe COVID-19 infection compared to the general population. An Italian study elucidated that the hospitalization rate for patients with cancer is approximately 56.6%, which is markedly higher than the 34.4% observed in the general population. Furthermore, patients with cancer exhibit a mortality rate of 14.7%, in contrast to 4.5% of healthy individuals ^(3)^. A comprehensive meta-analysis covering 52 studies reported a pooled case mortality rate of approximately 25.6% among patients with cancer infected with COVID-19 ^(4)^.

Specifically, patients with hematological malignancies, which constitute approximately 9% of all cancer diagnoses, are predisposed to severe SARS-CoV-2 infections because of immune system compromises ^(5)^. They have a 37% higher mortality risk when infected with COVID-19 and are prone to prolonged virus shedding and delayed seroconversion ^(6), (7)^. Given the aforementioned issues, it is crucial for these individuals to receive the COVID-19 vaccine. However, many clinical trials excluded these patients, leading to limited data on the vaccine efficacy of this group. Studies indicate that they have a reduced response to vaccines, putting them at risk of fatal COVID-19 infection and death ^(8), (9)^. Therefore, the Centers for Disease Control and Prevention (CDC) has approved an additional booster dose for all immunocompromised patients ^(10), (11)^.

This systematic review compared the vaccine efficacy between healthy adults and patients with hematological malignancies. A systematic review published in early 2022 reported the immunogenicity of seven types of hematological malignancies against the COVID-19 vaccine ^(12)^. Although some other studies have evaluated the seroconversion rate in these patients, very few have made direct comparisons with healthy controls ^(13), (14), (15)^. This review seeks to fill that knowledge gap by analyzing vaccine responses in patients with hematological malignancies in relation to healthy individuals.

## Materials and Methods

The present systematic review followed the Preferred Reporting Items for Systematic Reviews and Meta-Analysis checklist 2020 by Page et al. ^(16)^. The review protocol was registered with PROSPERO, the prospective international register of systematic reviews (CRD42022342545).

### Search strategies and selection criteria

A systematic literature search was conducted using four databases, including Medline via PubMed, EMBASE, Cochrane Library, and World Health Organization (WHO) COVID-19 Research Database. All searches included Medical Subject Headings (MeSH) terms and keywords, which were combined with the Boolean operators AND and OR (Table 1 and 2). No restrictions on the language of publication were applied. Articles from December 2020 to May 2022 were included. The references within these articles were also explored to reach the maximum number of related papers.

**Table 1.** Population, Intervention, Comparison, Outcome, and Study Types.

Characteristics	Inclusion	Exclusion
Population	Adult participants over 18 years with hematological malignancy and had received at least one dose of the COVID-19 vaccine.	Patients with any other diseases and/or any group other than healthy populations.
Intervention (Exposure)	First, second, or booster doses of COVID-19 vaccination	Nonvaccinated population
Comparison	Immune response to COVID-19 vaccination in patients with hematological malignancies and healthy control group. Immunoglobulin G (IgG) level, Neutralizing antibody (nAb) level	Any malignancy other than patients with hematological malignancies, i.e., solid tumor.
Outcome	Rates of seropositivity after one or two doses of COVID-19 vaccine, rates of positive nAb response after one or two doses of vaccine.	Side effects (if any) of the vaccines on the exposed and healthy group, cross-classification of the rates of seropositivity, and rates of positive nAb response with respect to other potential confounders.
Study type	All types of observational studies, experimental studies, and clinical trials.	Incomplete studies, studies not reporting any antibody level, and studies reporting CD4 and CD8 cell count in response to COVID-19 vaccination.

**Table 2.** Search Strategies.

Characteristics	Description
Databases	Medline via PubMed
	EMBASE
	WHO COVID-19 Research Database
	Cochrane Library
Other searches	Manual search using Keywords in Google Scholar
	Reference list from selected articles
Boolean operators	AND
	OR
Antibody or seroconversion-related keywords, MeSH terms	“Antibody formation”
	“Seroconversion”
	“Antibodies, neutralizing”
	“Neutralizing Antibodies”
	“seropositive*”
	“seroconversion*”
	“Antibody produce*”
	“Antibody response*”
	“Neutralizing antibody*”
Hematologic malignancy-related keywords, MeSH terms	“Primary Myelofibrosis”
	“Polycythemia Vera”
	“Myelodysplastic Syndromes”
	“Waldenstrom Macroglobulinemia”
	“Lymphoma”
	“Multiple Myeloma”
	“Multiple Myeloma”
	“Leukemia”
	“Leukemia”
	“Hematologic Neoplasms”
	“Hematologic neoplasm*”
	“Hematologic malignan*”
	“malignant*”
	“neoplasm*”
	“hematologic*”
	“hematologic*”
COVID-19-related keywords, MeSH terms	“Vaccination”
	“COVID-19 vaccines/administration and dosage”
	“COVID-19 vaccines/immunology”
	“SARS-COV-2 VACCINES”
	“COVID-19 VACCINES”

Initially, the title and abstract were carefully checked for preliminary screening. In the second stage of screening, Rayyan QCRI, an intelligent systematic review tool for literature screening developed by Ouzzani and Hammady, was used ^(17)^. The inclusion criteria comprise all completed randomized controlled trials, quasi-experimental studies, and observational studies. Studies assessing the efficacy of the COVID-19 vaccine in healthy individuals versus those with hematological malignancies were considered. Selected studies had at least one reported neutralizing antibody defined as seroconversion and the seroconversion rates of both groups after COVID-19 vaccination.

Studies lacking sufficient details on target populations and outcomes or not enabling effect size calculation (e.g., no data on the means and standard deviations for the patient and control groups, respectively) were excluded. The review excluded case reports, review papers, nonacademic publications such as editorials, and conference proceedings. The specific criteria of the population, intervention, comparison, outcomes, and study types are summarized in Table 1.

Two authors (ADB and ELAM-T) conducted an independent screening of the titles and abstracts of relevant papers and assessed the eligibility of full-text articles. Disagreements were resolved through discussion and consensus and were finally checked. Finally, articles for which the full text was inaccessible were eliminated and the records of these excluded articles were maintained in a supplementary document.

### Data extraction

Data were extracted independently by two authors (ADB and ELAM-T) using a Microsoft Office spreadsheet. Extracted information included study information (first author, year, country, and study design), population (sample characteristics, time points, hematological malignancy type, mean age in years, and % of male), exposure (vaccine, dose interval, and dose administered), comparison (comparison group, mean age in years, and % of male), and outcome (seroconversion rate and seroconversion cutoff value). A recheck was conducted for any unavailable data from a particular study. Mendeley (reference management software) was used for data management and references.

### Data synthesis and analysis

The findings of this review were presented in a narrative form. The Mantel–Haenszel method was used to estimate the pooled risk ratio and the corresponding 95% confidence interval for the outcome of interest. The hypothesis was tested using the *Z* statistic (level of significance p < 0.05). Between-study heterogeneity was estimated using Cochran’s Q and *I^2^* indices. The chi-square test was used to test statistical heterogeneity (*I^2^*) ^(18)^. Random-effects meta-analysis was performed under the appearance of statistical heterogeneity. The between-study variance (τ^2^) in the random-effects model was estimated using the der Simonian–Laird estimator. The risk ratio (RR) was calculated as a relative effect, along with a 95% confidence interval (CI). All of the results of the meta-analysis were presented in a forest plot. Two different meta-analyses were performed: one for the outcome after the first dose and another for the outcome after the second dose. All analyses were performed using the programing language R ^(19)^.

### Risk of bias assessment

The risk of bias for each study was assessed using the Newcastle–Ottawa scale for cohort studies ^(20)^. Each study was classified as low/intermediate/high risk of bias on the basis of the score obtained in three domains, namely, selection, comparability, and outcome domains. The three domains include eight criteria. Scores ≥7–9, 4–6, and < 4 are considered low, intermediate, and high risks of bias study, respectively.

### Publication bias and sensitivity analysis

The publication bias of the selected studies was checked using qualitative and quantitative methods. A funnel plot was used as a qualitative tool to check the publication bias through visual inspection. Another quantitative method was used to assess publication bias, that is, Egger’s test ^(21)^.

## Results

**Figure 1.**
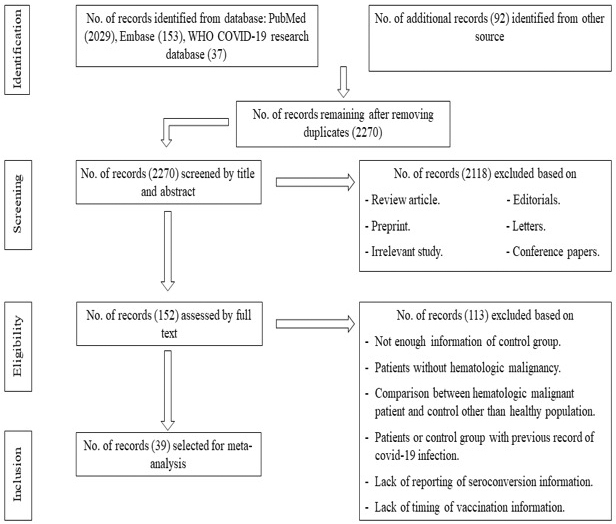
Flowchart of the article selection process.

From the initial 2,311 studies, 2,029 came from PubMed; 153, from EMBASE; 37, from the WHO COVID-19 Research Database; and 92, from other sources. Our initial stage of the search included 2,311 studies, of which 2,029 were from PubMed; 153, from EMBASE; 37, from the WHO COVID-19 Research Database; and 92, from other sources, i.e., manual searches and reference lists from literature (Figure 1). After removing 41 duplicates and screening titles, 2,118 articles were excluded. Of the 152 articles assessed in full, 113 were discarded because of not meeting the inclusion criteria. Finally, 39 articles representing approximately 10,854 patients were selected for this systematic review and meta-analysis. The study selection process is detailed in Figure 1.

After the completion of the full screening, 39 studies were selected ^(8), (22), (23), (24), (25), (26), (27), (28), (29), (30), (31), (32), (33), (34), (35), (36), (37), (38), (39), (40), (41), (42), (43), (44), (45), (46), (47), (48), (49), (50), (51), (52), (53), (54), (55), (56), (57), (58), (59)^ for our final analysis. Most of the studies were prospective cohort studies that included a comparison between seroconversion rates in both the patient group and control group (Table 3). Different studies used different cutoff values for seropositivity. The population characteristics of all studies are included in Table 3.

Furthermore, various types of hematological malignancies were addressed by the different studies. Multiple myeloma and chronic lymphocytic leukemia were the most concerning malignancies evaluated by 12 and 10 studies, respectively. Of the 39 studies, 8 were conducted in the USA; 6, in the UK; 5, in Italy; 7, in Israel; 5, in Greece; 3, in France; 2, in Belgium; and 1 each, in Austria, Australia, and Sweden. In addition, 34 out of the 39 studies were published in 2021, and the rest in 2022.

### Study outcome

Data on the proportion of male and female patients had been compiled from 38 studies. However, data on the gender distribution in the control group were available from only 20 studies. There were 45% females and 55% males in the patient group. By contrast, 58% of females and 42% of males were identified in the control group.

Of the 39 studies, 17 assessed the immunogenicity of both BNT162b2 and mRNA-1273; 11 assessed the immunogenicity of BNT162b2; 7 assessed the immunogenicity of both BNT162b2 and AZD1222; 2 assessed the immunogenicity of BNT162b2, mRNA-1273, and Ad26.COV2. S; and 2 assessed the immunogenicity of BNT162b2 and ChAdOx1, BNT162b2, mRNA-1273, and AZD1222.

Among the 39 studies, 14 focused on the seroconversion rate after the first dose and 35 included information on the second dose. Ten studies reported seroconversion after both the first and second doses. This review represents the seroconversion rates according to the type and dose of the vaccine. The BNT162b2 and ChAdOx1 vaccine combination resulted in the highest seropositivity, peaking rate at approximately 70% after the completion of both doses. However, BNT162b2 and mRNA-1273 also achieved similar seropositivity rates.

Multiple myeloma, chronic lymphocytic leukemia, and lymphoma were the predominant concerns among those studies. In total, 15 studies reported seropositivity among patients with multiple myeloma, which was substantially lower than that of the healthy controls. Among these patients, the highest seroconversion rate was 89% after the second dose. A total of 10 studies provided information on CLL, whereas 12 studies discussed lymphoma. The seropositivity rates reported in each study included in this systematic review are shown in Table 3. The highest seropositivity rates in CLL and lymphoma were 75% and 71%, respectively, which were less than the seroconversion rates achieved by patients with multiple myeloma. Conversely, several studies on these malignancies reported 98%–100% seropositivity among control groups.

**Table 3.** Population Characteristics of the Included Studies.

Study information	Population			Exposure			Comparison	Outcome		Risk of bias
First Author, Year, Country, Study design	Seroconversion in patients’ group (S.R-Seroconversion rate)	Hematological malignancy type	Age in years, % of male	Vaccine	Dose interval	Dose administered	Comparison group, age in years, % of male	Seroconversion in healthy controls (S.R-Seroconversion rate)	Seroconversion cutoff value	
Aleman et al., 2021, USA, Prospective cohort	Dose 2:	MM	Mean [range]: 65 [47–79], 63%	BNT162b2, mRNA-1273	28D or 21D	Second Dose	Healthy participants (n = 12), Mean [range]: 59 [45–64], 50%	12	≥3.2 AU/mL	7
	Patients (n = 44)							(n = 12)		
	Seronegative (n = 27)							S.R-100%		
	Seropositive (n = 17)									
	S.R-39%									
Avivi et al., 2021, Israel, Prospective cohort	Dose 2:	MM, SM	Median [range]: 70 [38–94], 56%	BNT162b2, mRNA-1273	21D	Second Dose	Median [range]: 67 [41–84], 42.2%	63	≥0.8 IU/mL	7
	Patients (n = 171)							(n = 64)		
	Seronegative (n = 38)							S.R-98%		
	Seropositive (n = 133)									
	S.R-78%									
Bergman et al., 2021, Sweden, Prospective cohort	Dose 2:	CLL	NA	BNT162b2, mRNA-1273	21D	Second Dose	NA	69	≥0.8 U/mL	9
	Patients (n = 388)							(n = 2)		
	Seronegative (n = 108)							S.R-95.8%		
	Seropositive (n = 280)									
	S.R-72%									
Bitoun et al., 2021, France, Retrospective cohort	Dose 2:	MM	Median [range]: 71 [46–93], 44%	BNT162b2, mRNA-1273	28D	Second Dose	Median [range]: 58 [26–88], 28.6%	26	≥0.4 IU/mL	5
	Patients (n = 27)							(n = 28)		
	Seronegative (n = 3)							S.R-96%		
	Seropositive (n = 24)									
	S.R-89%									
Canti et al., 2021, Belgium, Prospective cohort	Dose 2:	alloHCT	Median [range]: 60 [26–76], 47.5%	BNT162b2, mRNA-1273	21D	Second Dose	Median [range]: 48 [23–64], 27.5%	40	≥5 IU/mL	8
	Patients (n = 37)							S.R-88%		
	Seronegative (n = 5)									
	Seropositive (n = 32)									
	S.R-86%									
Chowdhury et al., 2021, UK, Retrospective cohort	Dose 1:	CML, ET, PV, MF, MDS	Median [IQR]: 62 [52–73], 45.8%	BNT162b2, AZD1222	2 W	First Dose	Median [range]: 62 [60–76]	224	≥50 AU/mL	6
	Patients (n = 59)							(n = 232)		
	Seronegative (n = 25)							S.R-97%		
	Seropositive (n = 34)									
	S.R-58%									
Chung et al., 2021, USA, Prospective cohort	Dose 1:	leukemia, lymphoma, MM	Median [range]: 65 [22–97], 56.4%	BNT162b2, mRNA-1273	28D or 21D	First Dose and Second Dose	Median [range]: 31 [22–67]	First Dose: 59	Immunoassay ≥50.0 AU/mL	6
	Patients (n = 167)							(n = 59)		
	Seronegative (n = 81)							S.R-100%		
	Seropositive (n = 86)							Second Dose: 54		
								(n = 54)		
	Dose 2:							S.R-100%		
	Patients (n = 456)									
	Seronegative (n = 142)									
	Seropositive (n = 314)									
	S.R-51% & 69%									
Crombie et al., 2021, USA, Prospective cohort	Dose 1:	Lymphoma and CLL	Median [range]: 69 [30–82], 43.5%	BNT162b2, mRNA-1273	28D or 21D	First Dose and Second Dose	Median [range]: 24 [22–56], 43.5%	First Dose: 23	1.07	5
	Patients (n = 22)							Second Dose: 23		
	Seronegative (n = 13)									
	Seropositive (n = 9)									
	
	Dose 2:									
	Patients (n = 21)									
	Seronegative (n = 9)									
	Seropositive (n = 12)									
	S.R-41% & 57%									
Fiorino et al., 2021, Italy, Prospective cohort	Dose 2:	MF	Median [range]: 67 [31–85], 50%	BNT162b2, mRNA-1273	3–4 W	Second Dose	NA	40	≥30%	7
	Patients (n = 42)							(n = 40)		
	Seronegative (n = 10)							S.R-100%		
	Seropositive (n = 32)									
	S.R-76%									
Gastinne et al., 2022, France, Prospective cohort	Dose 2:	lymphoma, leukemia	Median [range]: 62 [21–79], 60%	BNT162b2	28D	Second Dose	NA	25	≥0.8 U/mL	6
	Patients (n = 20)									
	Seronegative (n = 14)									
	Seropositive (n = 6)									
	S.R-30%									
Gavriatopoulou et al., 2021, Greece, Prospective cohort	Dose 1:	WM, CLL, NHL	Median [IQR]: 75 [40–88], 48.3%	BNT162b2, AZD1222	21D-3M	First Dose	NA	114	≥30% = positive, ≥50% = clinically relevant inhibition)	8
	Patients (n = 58)							(n = 232)		
	Seronegative (n = 50)							S.R-54%		
	Seropositive (n = 8)									
	S.R-14%									
Gavriatopoulou et al., 2021, Greece, Prospective cohort	Dose 1:	WM	Median [IQR]: 73 [64–81], 43.4%	BNT162b2, AZD1222	21D-3M	First Dose and Second Dose	Median [IQR]: 66 [62–82], 46.2%	First Dose: 212	≥30% = positive, ≥50% = clinically relevant inhibition)	8
	Patients (n = 106)							Second Dose: 212		
	Seronegative (n = 70)							S.R-96.2%		
	Seropositive (n = 36)									
	
	Dose 2:									
	Patients (n = 74)									
	Seronegative (n = 29)									
	Seropositive (n = 45)									
	S.R-34% & 61%									
Ghione et al., 2021, USA, Prospective cohort	Dose 2:	lymphoma	Median [range]: 70 [35–91], 52.3%	BNT162b2, mRNA-1273 and Ad26.COV2.S	28D or 21D	Second Dose	NA	154	≥1.0	6
	Patients (n = 86)							(n = 154)		
	Seronegative (n = 50)							S.R-100%		
	Seropositive (n = 36)									
	S.R-42%									
Guglielmelli et al., 2021, Italy, Prospective cohort	Dose 1:	MF, ET, PV	NA	BNT162b2, AZD1222	28D or 21D	First Dose	NA	14	≥15 AU/mL	6
	Patients (n = 30)							(n = 14)		
	Seronegative (n = 12)							S.R-100%		
	Seropositive (n = 18)									
	S.R-60%									
Herishanu et al., 2021, Israel, Prospective cohort	Dose 2:	CLL	Median [IQR]: 69.0 [63.0–73.7], 67.1%	BNT162b2	21D	Second Dose	Median [IQR]: 68 [64–74.7]	52	≥0.8 IU/mL = positive	8
	Patients (n = 52)							(n = 52)		
	Seronegative (n = 25)							S.R-100%		
	Seropositive (n = 27)									
	S.R-52%									
Jurgens et al., 2021, USA, Prospective cohort	Dose 2:	Lymphoma and CLL	Median [range]: 71 [24–90], 53.7%	BNT162b2, mRNA-1273	28D or 21D	Second Dose	NA	35	10000	5
	Patients (n = 67)									
	Seronegative (n = 36)									
	Seropositive (n = 31)									
	S.R-46%									
Lim et al., 2021, UK, Prospective cohort	Dose 1:	lymphoma	Median [IQR]: 69 [57–74], 62.8%	ChAdOx1, BNT162b2	10-12 W	First Dose and Second Dose	Median [IQR]: 45 [34–47], 33.3%	150	Meso scale discovery >0.55 BAU/mL, Anti–SARS-CoV-2 RBD IgG >0.73 BAU/mL	6
	Patients (n = 59)							(n = 150)		
	Seronegative (n = 27)							S.R-100%		
	Seropositive (n = 32)									
	
	Dose 2:									
	Patients (n = 86)									
	Seronegative (n = 25)									
	Seropositive (n = 61)									
	S.R-54% & 71%									
Mairhofer et al., 2021, Austria, Prospective cohort	Dose 2:	Cancer	NA	BNT162b2, mRNA-1273	28D or 21D	Second Dose	NA	28	≥50 IU/mL	7
	Patients (n = 45)							(n = 29)		
	Seronegative (n = 19)							S.R-96.6%		
	Seropositive (n = 26)									
	S.R-58%									
Malard et al., 2021, France, Retrospective cohort	Dose 2:	LM, MM	Median [range]: 68.9 [21.5–91.7], 59.7%	BNT162b2	28D	Second Dose	NA		≥50 AU/mL = positive; ≥3100 = neutralization)	9
	Patients (n = 196)									
	Seronegative (n = 105)									
	Seropositive (n = 91)									
	S.R-46%									
Marasco et al., 2022, Italy, Prospective cohort	Dose 2:	LM	Median [range]: 56 [46–62], 56.3%	BNT162b2, mRNA-1273	28D or 21D	Second Dose	Median [range]: 56 [46–62], 56.3%	167	≥0.8 IU/mL	9
	Patients (n = 167)							(n = 167)		
	Seronegative (n = 60)							S.R-100%		
	Seropositive (n = 107)									
	S.R-64%									
Marchesi et al., 2022, Italy, Prospective cohort	Dose 2:	MM	NA	BNT162b2	21D	Second Dose	NA	28	≥15 U/m	6
	Patients (n = 49)							(n = 28)		
	Seronegative (n = 12)							S.R-100%		
	Seropositive (n = 37)									
	S.R-76%									
McKenzie et al., 2021, UK, Prospective cohort	Dose 2:	HM	Median [IQR]: 35 [27–48], 64.7%	BNT162b2, mRNA-1273	70D	Second Dose	Median [IQR]: 66 [52.75–73], 69.2%	22	antibody level ED50, nAb assay: IC50	5
	Patients (n = 51)							(n = 26)		
	Seronegative (n = 29)							S.R-88%		
	Seropositive (n = 22)									
	S.R-43%									
Monin et al., 2021, UK, Prospective cohort	Dose 1:	Hematological Cancer	Median [IQR]: 73 [64.5–79.5], 52.3%	BNT162b2	21D	First Dose and Second Dose	Median [IQR]: 40.5 [31.3–50], 52.9%	First Dose: 32	≥70 EC50 = positive	7
	Patients (n = 44)							(n = 34)		
	Seronegative (n = 36)							S.R-94%		
	Seropositive (n = 8)							Second Dose: 12		
								(n = 12)		
	Dose 2:							S.R-100%		
	Patients (n = 5)									
	Seronegative (n = 2)									
	Seropositive (n = 3)									
	S.R-18% & 60%									
Parry et al., 2022, UK, Prospective cohort	Dose 2:	B cell CLL	Median [IQR]: 67 [60–72], 53%	AZD1222, BNT162b2	3 W	Second Dose	Median [IQR]: 71 [64–77], 55.9%	93	≥0.8	7
	Patients (n = 500)							(n = 93)		
	Seronegative (n = 165)							S.R-100%		
	Seropositive (n = 335)									
	S.R-67%									
Parry et al., 2021, UK, Prospective cohort	Dose 1:	CLL	Median [IQR]: 69 [63–74], 54.7%	AZD1222, BNT162b2	12 W and 3 W	First Dose and Second Dose	NA	First Dose: 95	≥0.8	7
	Patients (n = 86)							Second Dose: 59		
	Seronegative (n = 57)							(N = 95 & 59)		
	Seropositive (n = 29)							S.R-100%		
	
	Dose 2:									
	Patients (n = 12)									
	Seronegative (n = 3)									
	Seropositive (n = 9)									
	S.R-34% & 75%									
Peeters et al., 2021, Belgium, Prospective multicohort	Dose 2:	Cancer	Median [range]: 63 [25–79], 58.5%	BNT162b2	21D	Second Dose	NA	Second Dose: 40	≥5 IU/mL	6
	Patients (n = 41)							(n = 40)		
	Seronegative (n = 29)							S.R-100%		
	Seropositive (n = 12)									
	S.R-29%									
Perry et al., 2021, Israel, Prospective cohort	Dose 2:	B-NHL	Median [range]: 64 [20–92], 59.1%	BNT162b2	21D	Second Dose	Median [range]: 66 [25–83], 44.6%	65	≥0.8 IU/mL	8
	Patients (n = 149)							S.R-98.5%		
	Seronegative (n = 76)									
	Seropositive (n = 73)									
	S.R-49%									
Pimpinelli et al., 2021, Italy, Prospective cohort	Dose 1:	MM, MPM	Median [range]: 73 [47–78]	BNT162b2, mRNA-1273	21D	First Dose and Second Dose	Median [range]: 81 [79–87], 50%	First Dose: 36	≥15 U/m	9
	Patients (n = 92)		Median [range]: 70 [28–80], 53.3%					Second Dose: 36		
	Seronegative (n = 57)							n = 36		
	Seropositive (n = 35)							S.R-100%		
		
	Dose 2:									
	Patients (n = 92)									
	Seronegative (n = 15)									
	Seropositive (n = 77)									
	S.R-38% & 84%									
Rahav et al., 2021, Israel, Prospective cohort	Dose 2:	CLL, NHL, MM, and MDS	Median [IQR]: 69 [61–74]	BNT162b2	21D	Second Dose	NA	269	≥1.1	9
	Patients (n = 529)		Median [IQR]: 66 [59–73]					(n = 272)		
	Seronegative (n = 175)		Median [IQR]: 62 [49–70]					S.R-98.9%		
	Seropositive (n = 354)		Median [IQR]: 73 [66–80], 58.8%							
	S.R-67%									
Schiller-Salton et al., 2021, Israel, Prospective cohort	Dose 2:	MM	Mean ± SD: 67.8 ± 9.9, 58.50%	BNT162b2	30D	Second Dose	NA	Second Dose: 360	≥150 AU/mL	8
	Patients (n = 176)									
	Seronegative (n = 47)									
	Seropositive (n = 129)									
	S.R-73%									
Shem-Tov et al., 2022, Israel, Prospective cohort	Dose 2:	HSCT	Mean ± SD: 58 ± 14.0, 63.20%	BNT162b2, mRNA-1273	21D	Second Dose	Mean ± SD: 55.6 ± 14.2, 24.3%	269	Cut point: >1.1	7
	Patients (n = 152)							(n = 272)		
	Seronegative (n = 34)							S.R-98.9%		
	Seropositive (n = 118)									
	S.R-78%									
Shen et al., 2021, Australia, Prospective cohort	Dose 2:	CLL	Median [range]: 71.5 [22–94], 56.3%	BNT162b2, mRNA-1273, AZD1222	2-4 W	Second Dose	NA	25	≥50 AU/mL	6
	Patients (n = 160)									
	Seronegative (n = 72)									
	Seropositive (n = 88)									
	S.R-55%									
Stampfer et al., 2021, USA, Retrospective cohort	Dose 1:	MM	Median [range]: 68 [35–88], 59.4%	BNT162b2, mRNA-1273	28D or 21D	First Dose and Second Dose	Median [range]: 61 [26–85]	First Dose: 31	50-250 IU/mL partial response, >250 IU/mL	8
	Patients (n = 96)							Second Dose: 31		
	Seronegative (n = 76)							n-31		
	Seropositive (n = 20)									
								S.R-100%		
	Dose 2:									
	Patients (n = 96)									
	Seronegative (n = 32)									
	Seropositive (n = 64)									
	S.R-21% & 67%									
Terpos et al., 2021, Greece, Prospective cohort	Dose 1:	CLL, NHL, HL	Mean ± SD: 64.6 ± 14.3, 50%	BNT162b2	21D	First Dose and Second Dose	Mean ± SD: 69.8 ± 12.5, 44.9%	First Dose: 152	GenScript (≥30% = positive, ≥30% = clinically relevant)	6
	Patients (n = 132)							(n = 214)		
	Seronegative (n = 103)							S.R-71%		
	Seropositive (n = 29)							Second Dose: 214		
								(n = 214)		
	Dose 2:							S.R-98.1%		
	Patients (n = 132)									
	Seronegative (n = 65)									
	Seropositive (n = 67)									
	S.R-22% & 51%									
Terpos et al., 2021, Greece, Prospective cohort	Dose 1:	MM, SM, MGUS	Median [IQR]: 74 [62–80], 54.7%	BNT162b2, AZD1222	21D-3M	First Dose and Second Dose	NA	First Dose: 145	GenScript (≥30% = positive, ≥30% = clinically relevant)	8
	Patients (n = 276)									
	Seronegative (n = 159)							(n = 225)		
	Seropositive (n = 117)									
								S.R-64.2%		
	Dose 2:							Second Dose: 204		
	Patients (n = 276)							(n = 226)		
	Seronegative (n = 80)							S.R-90.3%		
	Seropositive (n = 196)									
	S.R-42% & 71%									
Terpos et al., 2021, Greece, Prospective cohort	Dose 1:	MM	Mean ± SD: 81.68 ± 7.63, 60.40%	BNT162b2	21D	First Dose	Mean ± SD: 81.56 ± 8.11, 54.8%	57	GenScript (≥30% = positive, ≥30% = clinically relevant)	8
	Patients (n = 48)							(n = 104)		
	Seronegative (n = 36)							S.R-54.8%		
	Seropositive (n = 12)									
	S.R-25%									
Thakkar et al., 2021, USA, Retrospective cohort	Dose 2:	HM	Median [range]: 67 [27–90], 42%	BNT162b2, mRNA-1273, Ad26.COV2.S	21D or 28D	Second Dose	NA	26	≥ 50 AU/mL	7
	Patients (n = 66)							(n = 26)		
	Seronegative (n = 10)							S.R-100%		
	Seropositive (n = 56)									
	S.R-85%									
Tzarfati et al., 2021, Israel, Prospective cohort	Dose 2:	HM	Median [IQR]: 70 [61–78], 55.9%	BNT162b2	21D	Second Dose	Median [IQR]: 69 [58–74], 43.5%	107	≥12 AU/mL positive)	8
	Patients (n = 315)							(n = 108)		
	Seronegative (n = 80)							S.R-99%		
	Seropositive (n = 235)									
	S.R-75%									
Van Oekelen et al., 2021, USA, Retrospective cohort	Dose 2:	MM	Median [range]: 68 [38–93], 58.1%	BNT162b2, mRNA-1273	28D or 21D	Second Dose	NA	67	5 AU/mL	8
	(n = 260)							(n = 67)		
	Seronegative (n = 41)							S.R-100%		
	Seropositive (n = 219)									
	84%									

It was found that there was significant heterogeneity in the meta-analysis of the effects of COVID-19 vaccination in patients with hematological malignancies for the first dose (*I^2^* = 80.6%, p < 0.0001) and for the second dose (*I^2^* = 87.8%, p < 0.0001). Between-study heterogeneity (τ2) for the first vaccine dose was (0.0844, p < 0.01). In the case of the second vaccine dose, between-study heterogeneity (τ2) was (0.0224, p < 0.01).

### First vaccine dose

**Figure 2.**
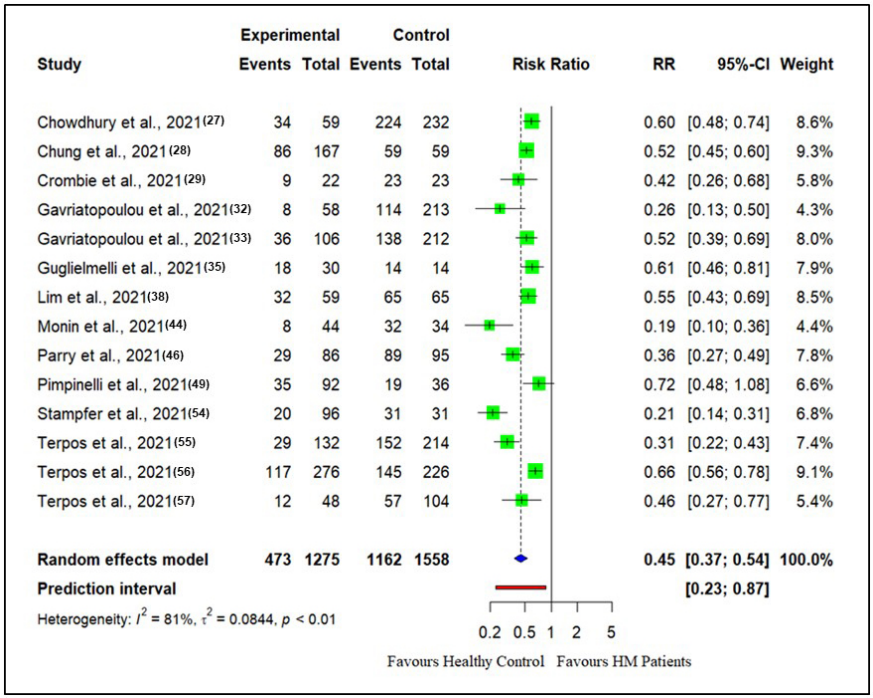
Risk ratios for seroconversion among patients with hematological malignancies compared with healthy controls after the first dose of the COVID-19 vaccine.

The RR for seroconversion rate after the first vaccine dose in patients with hematological malignancies compared with healthy controls is presented in Figure 2. There were 14 studies reporting seroconversion after the first vaccine dose in patients with hematological malignancies (n = 1275) compared with healthy controls (n = 1558). The seroconversion rates in patients with hematological malignancies (37.0%) were reduced compared with those in healthy controls (74.5%) after the first vaccine doses (RR: 0.45, CI (0.37, 0.54), Z = −8.78, p < 0.0001).

### Second vaccine dose

**Figure 3.**
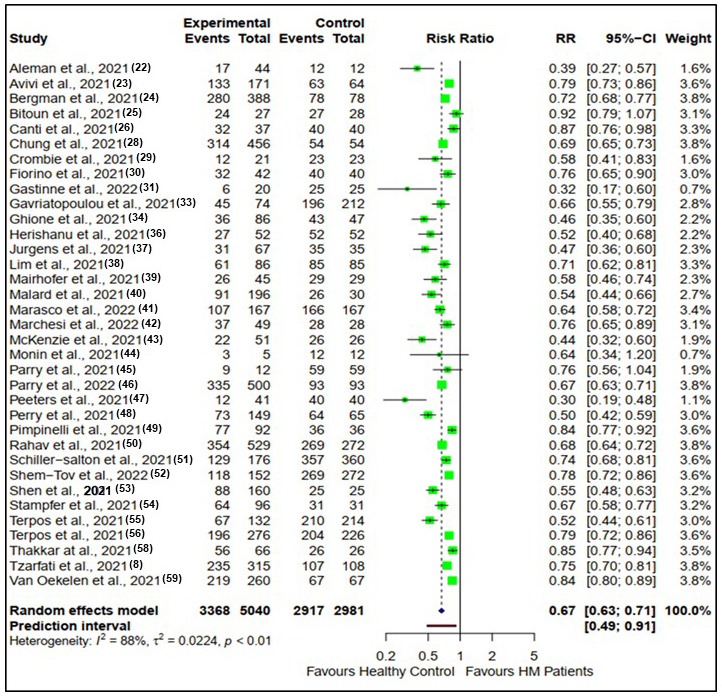
Risk ratios for seroconversion among patients with hematological malignancies compared with healthy controls after the second dose of the COVID-19 vaccine.

Similarly, after the second vaccine dose, the seroconversion rates for patients with hematological malignancies compared with healthy controls are presented in Figure 3.

There were 35 studies reporting seroconversion after the second vaccine dose in patients with hematological malignancies (n = 5040) compared with healthy controls (n = 2981). Seroconversion rates in patients with hematological malignancies (66.9%) were reduced compared with healthy controls (97.9%) after the second vaccine doses (RR: 0.67, CI (0.63, 0.71), Z = −13.56, p < 0.0001).

### Risk of bias assessment

As shown in Table 4, 25 studies were assessed to be at a low risk of bias and 14 at a moderate risk of bias. The risk of bias mainly represented the exposed cohort, with controls not being age-matched, lack of outcome information at the beginning, lack of follow-up length and adequacy of follow-up, and lack of available data at predetermined endpoints. The study list of the Newcastle–Ottawa scale for risk of bias assessment is presented in Table 4.

**Table 4.** Newcastle–Ottawa Scale for Risk of Bias Assessment of the Included Studies.

Study	Selection				Comparability	Outcome			Overall
	Representativeness of the exposed cohort	Selection of the nonexposed cohort	Ascertainment of exposure	Outcome not present at start		Assessment of outcome	Adequate follow-up length	Adequacy of follow up	
Aleman et al., 2021 ^(22)^	*		*	*	*	*	*	*	7
Avivi et al., 2021 ^(23)^	*		*	*	*	*	*	*	7
Bergman et al., 2021 ^(24)^	*	*	*	*	**	*	*	*	9
Bitoun et al., 2021 ^(25)^			*	*		*	*	*	5
Canti et al., 2021 ^(26)^	*	*	*	*	*	*	*	*	8
Chowdhury et al., 2021 ^(27)^	*		*	*		*	*	*	6
Chung et al., 2021 ^(28)^	*		*	*		*	*	*	6
Crombie et al., 2021 ^(29)^			*	*		*	*	*	5
Fiorino et al., 2021 ^(30)^	*		*		**	*	*	*	7
Gastinne et al., 2022 ^(31)^	*		*	*		*	*	*	6
Gavriatopoulou et al., 2021 ^(32)^	*	*	*	*	*	*	*	*	8
Gavriatopoulou et al., 2021 ^(33)^	*	*	*	*	*	*	*	*	8
Ghione et al., 2021 ^(34)^	*		*	*		*	*	*	6
Guglielmelli et al., 2021 ^(35)^	*		*	*		*	*	*	6
Herishanu et al., 2021 ^(36)^	*		*	*	**	*	*	*	8
Jurgens et al., 2021 ^(37)^	*		*			*	*	*	5
Lim et al., 2021 ^(38)^	*		*	*		*	*	*	6
Mairhofer et al., 2021 ^(39)^	*	*	*	*		*	*	*	7
Malard et al., 2021 ^(40)^	*	*	*	*	**	*	*	*	9
Marasco et al., 2022 ^(41)^	*	*	*	*	**	*	*	*	9
Marchesi et al., 2022 ^(42)^	*		*	*		*	*	*	6
McKenzie et al., 2021 ^(43)^			*	*		*	*	*	5
Monin et al., 2021 ^(44)^	*	*	*	*		*	*	*	7
Parry et al., 2022 ^(45)^	*		*	*	*	*	*	*	7
Parry et al., 2021 ^(46)^	*		*	*	*	*	*	*	7
Peeters et al., 2021 ^(47)^	*	*	*			*	*	*	6
Perry et al., 2021 ^(48)^	*		*	*	**	*	*	*	8
Pimpinelli et al., 2021 ^(49)^	*	*	*	*	**	*	*	*	9
Rahav et al., 2021 ^(50)^	*	*	*	*	**	*	*	*	9
Schiller-Salton et al., 2021 ^(51)^	*	*	*		**	*	*	*	8
Shem-Tov et al., 2022 ^(52)^	*		*		**	*	*	*	7
Shen et al., 2021 ^(53)^			*	*	*	*	*	*	6
Stampfer et al., 2021 ^(54)^	*	*	*		**	*	*	*	8
Terpos et al., 2021 ^(55)^	*	*	*	*	*	*	*	*	8
Terpos et al., 2021 ^(56)^	*	*	*	*	*	*	*	*	8
Terpos et al., 2021 ^(57)^	*	*	*			*	*	*	6
Thakkar et al., 2021 ^(58)^	*	*	*		*	*	*	*	7
Tzarfati et al., 2021 ^(8)^	*		*	*	**	*	*	*	8
Van Oekelen et al., 2021 ^(59)^	*	*	*		**	*	*	*	8

### Publication bias and sensitivity analysis

**Figure 4.**
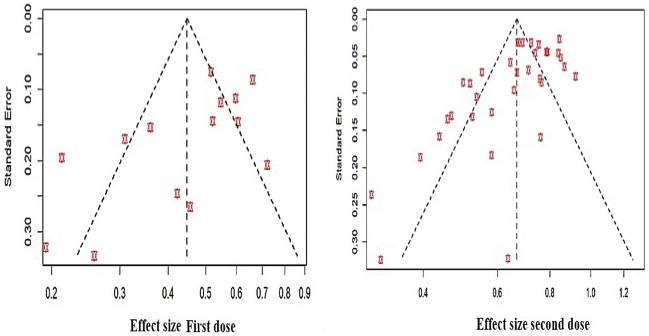
Funnel plot of the results for the first and second vaccine doses.

The results of publication bias assessment using a funnel plot are shown in Figure 4. On the basis of this assumption, the funnel plots for the first and second vaccine doses clearly show the presence of publication bias as they are highly asymmetrical.

## Discussion

The present systematic review and meta-analysis using 39 studies included several hematological malignancies for seroconversion. According to the findings of the present study, after the first vaccine dose, patients with cancer had an antibody response rate (37.0%) that was considerably lower than that of the healthy controls (74.5%). Following the second vaccine dose, the seroconversion rate in patients with cancer reached 66.8% whereas it peaked at 97.9% in the healthy controls. This result agreed with the findings of another meta-analysis of 27 studies that revealed seroconversion rates of 37.3%–51% and 57%–60% following the first and second vaccine doses, respectively, in the patient group ^(12)^. Patients with multiple myeloma displayed lower seroconversion rates compared with healthy individuals after receiving the COVID-19 vaccine. Out of 2000 patients with myeloma who were vaccinated, seropositivity rates after the first dose varied between 21.43% and 38%. This rate increased to 89% after the second dose. By contrast, the seroconversion rate increased to 89% following the second vaccine dose. However, a previous study found high seroconversion rates of 93% with myeloma therapy and 94% without myeloma therapy after vaccination ^(60)^. According to another study, 52% of individuals tested positive for SARS-CoV-2 IgG antibodies following the second vaccine dose, which was lower than that of our result ^(13)^. However, two of the studies that we reviewed reported active treatment to be associated with reduced response rates ^(13), (60)^. Factors influencing these variable rates in patients with myeloma include the extent of immunoparesis, relapsed cases, monoclonal antibody treatment, and renal failure, which can lead to vaccine inefficacy.

Thirteen studies focused on leukemia, with 10 highlighting the low seropositivity in patients with CLL after vaccination compared with the multiple myeloma group. CLL is less aggressive in stages A and B, requiring intensive treatment mostly in stage C. Because of treatments, especially in stages B and C, patients with CLL often experience relative immune suppression ^(61)^. In this review, only 51%–75% of patients with CLL showed an optimal humoral response after COVID-19 vaccination. This aligns with a prior study reporting a 52% seroconversion rate post-immunization ^(14)^. One study noted that patients on tyrosine kinase inhibitors had a 16.8% seroconversion rate, which was lower than the 26.8% reported in another systematic review and meta-analysis ^(62)^.

There were 12 reviewed studies that reported reduced seroconversion rates in patients with lymphoma. The seropositivity rates after the second dose ranged from 42% to 71%. This finding was consistent with the previous study, which observed an almost 60% seroconversion rate in patients ^(63)^. The same study reported a 69% seroconversion rate in patients with allogeneic hematological stem cell transplantation, whereas a considerably high (86.4%) seropositivity rate was found in one of our studies. Regarding vaccine efficacy, a prior study indicated that COVID-19 mRNA vaccines were highly protective in patients with cancer but had a lower efficacy than that of the healthy controls ^(64)^.

According to our study, the BNT162b2 and ChAdOx1 vaccine combination as the first and second doses achieved the highest seroprotection (70%) against COVID-19. Almost all vaccines provided a 98%–100% seroconversion rate in healthy controls. Our findings reaffirmed the efficacy of mRNA vaccines (BNT162b2) and viral vector vaccines (ChAdOx1). The mRNA vaccines stimulate helper T cells, producing neutralizing antibodies (nAbs) and developing immune memory, which enhances the protection against COVID-19. This makes BNT162b2 effective even in immunocompromised patients. ChAdOx1, using a nonreplicating adenovirus vector, helps the immune system recognize and combat SARS-CoV-2’s spike protein. This dual-pathway activation benefits patients with hematological malignancies. Taken together, the up-to-date evidence generated by this systematic review and meta-analysis on the antibody response in patients with hematological malignancies and the clinical efficacy of vaccines will aid in formulating a newer vaccine policy for these individuals.

### Limitations

The present review had several limitations. First, only seroconversion data were taken from studies for analysis without any adverse effect of COVID-19 vaccine data. Second, the cutoff points for seropositivity varied across studies. Although it must be considered that the values may differ slightly depending on the testing method or population, it is unlikely that this difference had a significant impact on the comparison between the two groups. Third, most of these studies did not include any prior history of SARS-CoV-2 infection data that might influence the seropositivity analysis. Fourth, the treatment options were not examined, which needs to be analyzed in more detail in the future. Lastly, the immune response to booster doses could not be evaluated because there was not enough information regarding seroconversion by the third and fourth COVID-19 vaccine doses.

## Conclusion

The present systematic review clearly illustrated that patients with hematological malignancies have lower antibody titers compared with healthy controls. Therefore, these patients should carefully adhere to COVID-19 preventive measures or should be given priority when receiving a booster dose of the vaccine. Although the response rates were inadequate, vaccination is still regarded as important and should be performed before the start of anticancer therapy whenever possible. Long-term self-protective measures such as mask use, sanitization, and avoidance of social contact are always required for these patients.

## Article Information

### Conflicts of Interest

None

### Author Contributions

ADB and ELAM-T conceived the research topic. ADB contributed to the collection of clinical information, data analysis, and manuscript preparation under the supervision of ELAM-T. All authors critically reviewed and revised the manuscript and approved the final version for submission.

### Approval by Institutional Review Board (IRB)

This systematic review used studies that are published in several medical databases. Ethics approval was not required for this study.
